# Associations of chronic liver disease and liver cancer with glyphosate and its metabolites in Thailand

**DOI:** 10.1002/ijc.35282

**Published:** 2024-12-09

**Authors:** Daxesh P. Patel, Christopher A. Loffredo, Benjarath Pupacdi, Siritida Rabibhadana, Panida Navasumrit, Jittiporn Chaisaingmongkol, Leila Toulabi, Majda Haznadar, Bhavik Dalal, Mohammed Khan, Joshua Stone, Vajarabhongsa Bhudhisawasdi, Nirush Lertprasertsuke, Anon Chotirosniramit, Chawalit Pairojkul, Chirayu U. Auewarakul, Thaniya Sricharunrat, Kannika Phornphutkul, Suleeporn Sangrajrang, Anuradha Budhu, Chulabhorn Mahidol, Xin W. Wang, Frank J. Gonzalez, Mathuros Ruchirawat, Curtis C. Harris, the TIGER‐LC Consortium

**Affiliations:** ^1^ Laboratory of Human Carcinogenesis, Center for Cancer Research National Cancer Institute Bethesda Maryland USA; ^2^ Georgetown University Medical Center Washington DC USA; ^3^ Chulabhorn Research Institute Bangkok Thailand; ^4^ Center of Excellence on Environmental Health and Toxicology, (EHT) OPS, MHESI Bangkok Thailand; ^5^ Khon Kaen University Khon Kaen Thailand; ^6^ Chiang Mai University Chiang Mai Thailand; ^7^ Chulabhorn Hospital Bangkok Thailand; ^8^ Rajavej Hospital Chiang Mai Thailand; ^9^ National Cancer Institute Bangkok Thailand; ^10^ Laboratory of Metabolism, Center for Cancer Research, National Cancer Institute National Institutes of Health Bethesda Maryland USA

**Keywords:** chronic liver disease, environmental carcinogens, glyphosate, hepatocellular carcinoma, hospital‐based case control study

## Abstract

Glyphosate [N‐(phosphonomethyl) glycine], a systemic herbicide, is used globally (825 million kg/year) in 750+ formulations. The International Agency for Research on Cancer classified glyphosate is a probable human carcinogen (Group 2A), but epidemiological studies have been lacking for its association with liver cancer and chronic liver disease. We analyzed urine specimens from 591 patients with newly diagnosed liver cancer, chronic liver disease (CLD), and healthy individuals from five different medical centers between 2011 to 2016 in Thailand. Gas chromatography electrospray ionization mass spectrometry (GC‐ESI/MS) was used to quantify glyphosate and its metabolites, aminomethylphosphonic acid (AMPA) and phosphoric acid (PPA) to study their levels in urine of hepatocellular carcinoma (HCC) and CLD patients in comparison to matched healthy individuals. Significantly higher levels of glyphosate were found in CLD patients compared to HCC cases and hospital controls, while significantly elevated levels of both AMPA and PPA were observed in HCC and CLD patients compared to hospital controls. Glyphosate and its metabolites were also detected at low to moderately high levels in convenience samples of food products and drinking water. These results raise concerns about the potential role of glyphosate in chronic liver disease and liver cancer risk.

## Graphical abstract

What's new?

The IARC classifies the herbicide glyphosate as a probable carcinogen, and animal studies have shown that glyphosate exposure harms the liver and kidneys. Here, the authors investigate the relationship between glyphosate exposure and chronic liver disease (CLD) and hepatocellular cancer (HCC). They analyzed urine samples from patients newly diagnosed with HCC, those with CLD, and healthy individuals for the presence of glyphosate and its metabolites. CLD patients had higher levels of glyphosate compared with HCC patients and controls, and glyphosate metabolites were present at higher levels in both HCC and CLD patients compared with controls.

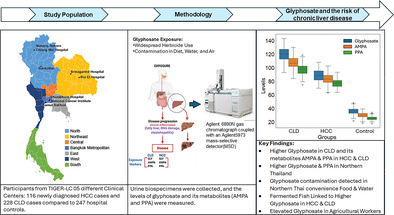

## 1 INTRODUCTION

Chronic liver disease is an important cause of morbidity and mortality worldwide. CLD patients are more likely to develop liver fibrosis, cirrhosis, and cancer, predominantly hepatocellular carcinoma (HCC).^1^ Hepatitis B (HBV) and C (HBC) chronic infections, chemical carcinogens, alcohol‐related liver disease, and non‐alcoholic fatty liver disease (NAFLD) are the most common etiologies of both CLD and HCC.^1^ NAFLD and its advanced stage, non‐alcoholic steatohepatitis (NASH), are of major concern due to their increasing global health burden.^1^, ^2^

Emerging research on glyphosate and AMPA exposures suggests that these chemicals disrupt lipid and glucose metabolism, and can lead to hepatic steatosis, inflammation and other abnormalities in the liver.^3^, ^4^ Glyphosate‐based herbicides (GBH), are the most commonly used herbicides worldwide.^5^, ^6^ GBH residues are routinely found in food and drinking water.^5^, ^7^ Although epidemiological research on GBH residues in the human body is limited, recent evidence suggests that exposures to glyphosate and its metabolites are widespread.^8^ Several animal studies have demonstrated that glyphosate exposure induces toxic effects on liver and kidney function.^5^, ^9^, ^10^, ^11^ Although these studies used dosages higher than the acceptable daily intake for humans, the observed hepato‐renal toxicity in animal models suggests a potential risk that warrants further investigation.^6^, ^12^

The International Agency for Research on Cancer classifies glyphosate as a probable (Group 2A) human carcinogen, based on epidemiological associations of chronic, occupational exposure with an increased risk of non‐Hodgkin's lymphoma (NHL).^13^ Recent studies have also identified a consistent link between glyphosate exposure and an elevated risk of follicular lymphoma.^14^, ^15^ However, results remain inconclusive, as meta‐analyses of existing reports show mixed findings, with some studies suggesting no significant association,^16^ although this study has been criticized for potential methodological limitations.^13^, ^17^ Further research is needed to clarify the relationship between glyphosate and lymphoid malignancies. Data on glyphosate and HCC risk has been limited to date, but accumulating evidence from animal studies shows that glyphosate generates reactive oxygen species and causes chronic inflammation and transcriptomic changes in the liver.^11^ Our objective is to fill gaps in knowledge on associations of glyphosate with CLD and HCC in our ongoing hospital‐based case control study in Thailand,^18^ where a substantial proportion of the population works in the agricultural sector and is exposed to high levels of glyphosate.^18^, ^19^ Urine samples were obtained from 591 participants across five clinical centers (Figure S1A) to investigate potential associations of glyphosate and its major metabolites (Figure S1B) with HCC and CLD.

## 2 MATERIALS AND METHODS

### 2.1 Overall hospital‐based case control study design

This report is based on a larger study, the Thailand Initiative in Genomics and Expression Research for Liver Cancer (TIGER‐LC), which is a consortium between the US National Cancer Institute and the Chulabhorn Research Institute in Thailand, formed in 2010 to explore genetic, environmental, and epigenetic risk factors for liver malignancies, which are highly prevalent in Thailand.^20^ TIGER‐LC is a hospital‐based case control study, with two sets of case groups (HCC and CLD: the latter as a high‐risk disease that often leads to HCC) and a pool of healthy hospital controls.^18^

Cases and hospital controls were recruited at the clinical centers described below, and participants provided a urine sample and questionnaire information as detailed below. Prevalent cases were not included; only newly diagnosed cases were enrolled in the study on the day they reported to the hospital for trans‐arterial chemoembolization (TACE), chemotherapy, or surgery. The response proportions of subjects who were approached to be in the study but declined to participate were 7.8% of HCC cases, 15.8% of CLD patients, and 1.1% of hospital controls. There were 591 participants in our analysis.

### 2.2 Participating clinical centers

Five clinical centers participated in recruiting participants (Figure S1A). Chiang Mai University Hospital recruited subjects in the northern part of Thailand. Chulabhorn Hospital and the Thailand NCI Hospital recruited mainly in Bangkok and the central region of the country. Khon Kaen University Hospital and Roi Et Hospital recruited patients in the northeast. The coordinating center was Chulabhorn Research Institute in Bangkok.

### 2.3 Enrollment of HCC and CLD cases

HCC cases were defined as persons with confirmed primary malignancy of the liver based on pathology, liver mass imaging results from magnetic resonance imaging (MRI), computed tomography (CT) or ultrasound (US), and serum levels of alpha‐fetoprotein (AFP). The HCC classification for subjects without a biopsy‐confirmed result included the following criteria: (a) one positive imaging test result and AFP levels of 200 or higher; (b) one imaging test and one histology result; or (c) two different imaging modalities if the tumor was 2 cm or larger, with these imaging modalities used to exclude metastatic tumors based on gross morphology. They were recruited from the liver surgery services at the participating hospitals. At Roi Et Hospital, cases were also recruited from the ultrasonography screening clinic if they met all the inclusion criteria listed above. CLD cases were selected based on chronic HBV and/or HCV infections or alcoholic liver disease, with no history of liver cancer, and were recruited at the hepatology units of the same hospitals as the HCC patients, and CLD patients and hospital controls were matched to the HCC cases by sex and age (within 5 years), separately in each recruitment hospital.

### 2.4 Recruitment of hospital controls

Hospital controls were recruited from the general health check‐ups department at all centers except for Roi Et Hospital, which is near Khon Kaen University (Srinagarind) Hospital and shares the same catchment area. Khon Kaen hospital controls were therefore recruited as a comparison group for cases at both Roi Et Hospital and Khon Kaen University Hospital. Hospital controls were defined as persons without a history of cancer who were visiting the participating hospitals for wellness checkups and other routine procedures. They were free of HCV and HBV, were not relatives of cases, and fit within the age (5‐year age groups), sex, and geographic (central, north, northeast, and other regions) strata required for frequency‐matching to the case groups.

In addition, Khon Kaen University Hospital conducted recruitment drives in rural villages within its catchment area using the same inclusion and exclusion criteria, and at the Khon Kaen site only, there are periodic health surveillance visits by a team of doctors and nurses from the university hospital to the nearby rural areas, where residents are invited to be screened for liver flukes in the stools. Those who tested negative were eligible to be controls for our study.

### 2.5 Interview and collection of biospecimens

Nurse‐administered interviews, conducted in either the hospital or a rural health clinic using a structured questionnaire to gather self‐reported data on demographics, medical history, lifestyle and environmental exposures, and diet, were carried out without offering any incentives to the participants. Specific variables are defined below. 50 ml of midstream urine samples were collected from each participant, aliquoted into 15 ml tubes, and stored at −80°C. There were no restrictions on fasting/feeding or dietary/drug intake. Aliquots were transferred to the coordinating center at Chulabhorn Research Institute. Selected aliquots were shipped to the US National Cancer Institute (NCI) Laboratory of Human Carcinogenesis for herbicide exposure analysis.

### 2.6 Data management

Completed questionnaires were delivered to the coordinating center for quality control and data entry. Data from questionnaires were entered manually into a database system, and visual confirmation of the entered data by side‐by‐side comparisons was performed against the paper forms. Quality control steps ensured data integrity and correctness, including logical error checking.

### 2.7 Analytes quantitation

Glyphosate and its metabolites (AMPA and PPA) in urine were quantified by gas chromatography‐electrospray ionization/mass spectrometry (GC‐ESI/MS) using DL‐norleucine as an internal standard. Chemicals (purity >99%) were from MilliporeSigma, with MTBSTFA +1% TBDMCS from Regis Technologies. Urine samples, prepped with DLN and urease, were incubated at 60°C, then centrifuged, dried, and derivatized. An Agilent 6890 N GC coupled with an Agilent 5973 mass‐selective detector provided chromatographic separation. Calibration standards ranged from 10.0 to 10,000 nM. The GC method used a temperature ramp from 100°C to 300°C at 20°C/min. Detection was performed in SIM mode with retention times: Glyphosate (12.7 min, m/z 454.2), AMPA (11.2 min, m/z 396.2), and PPA (10.4 min, m/z 383.2). Due to the length of the analytical run, 75 samples were processed per day in a continuous inter‐batch procedure. In this study, running quality control (QC) samples between runs was not feasible because our focus was on measuring glyphosate and its metabolites in human samples. Since these analytes are exogenous, creating QC samples from pooled human urine that accurately reflected their concentrations was challenging. To avoid matrix effect profiling, we did not use aqueous standards as QC samples. Additionally, to prevent analytical variation for glyphosate and PPA, the dilution integrity factor was omitted during initial sample preparation. This approach initially led us to exclude total 490 AMPA samples (82.9% of the total: 83% of hospital controls, 76.7% of HCC, and 86% of CLD) below the lower detection limit, ensuring accurate concentration measurements and minimizing potential errors.

### 2.8 Variables

The outcome variables for this study are the urinary measurements of glyphosate and its two major metabolites, AMPA and PPA. Potential confounding variables included sex, region of current residence (north, northeast, central, or other), occupation (agricultural vs. non‐agricultural), HBV and HCV infection status (currently infected vs. not currently infected, as determined from HBsAG and HCV‐DNA tests, respectively), and alcohol drinking history (ever vs. never, and frequency of consuming alcohol for ever‐drinkers).

### 2.9 Statistical analysis

Experimental values are presented as mean ± SD. Study variables include concentrations of urinary glyphosate, AMPA, and PPA; sex, age at recruitment, and region of the country; body mass index (weight in kilograms divided by height in meters squared); self‐reported occupation (agricultural vs. non‐ agricultural sectors), personal herbicide use, and dietary variables. Statistical comparisons of the herbicide concentrations in urine were determined by Mann–Whitney *t*‐tests or two‐way ANOVA or Multiple T‐tests between two groups, and by Kruskal–Wallis's comparisons test among multiple groups. Multivariate analysis used unconditional linear regression with adjustment for age, gender, agricultural versus non‐agricultural occupation, region, ever versus never drinker, HBV and HCV (positive vs. negative for each virus) and consumption of raw or fermented fish. Linear and logistic regression analyses were applied to study the associations of HCC and CLD, separately, in comparison to hospital controls, Glyphosate, AMPA, and PPA were analyzed as continuous variables, quartiles (based on their distributions in hospital controls), and as dichotomized at the median value of the control distributions. We used Prism version 9.3.1 (GraphPad Software, San Diego, CA). Trends in odds ratios were evaluated using the chi‐square test for linear trend (https://epitools.ausvet.com.au/trend). *p* values <.05 were considered statistically significant.

## 3 RESULTS

### 3.1 Participants and overall comparisons of urinary pesticide levels in cases and hospital controls

We analyzed 591 urine samples from the participants (247 hospital controls, 116 HCC, and 228 CLD) for this study using gas‐chromatography mass spectrometry technique. Demographic information, including the mean ages, distributions by sex, region of current residence, occupation, and HBV and HBC infections are shown in Table 1, together with overall levels of glyphosate, AMPA, and PPA. Mean ages were similar between HCC cases and hospital controls (54–59 years), while CLD patients were somewhat younger (48 years). Males predominated across all groups, as expected. Chronic HBV and HCV were observed in nearly half of HCC cases, and in the majority of CLD.

**TABLE 1:** Demographic characteristics of TIGER‐LC participants.

TIGERLC all clinical centers	Hospital controls	CLD cases	HCC cases
Total number of samples	247	228	116
Mean age (SD)	53.5 (9.7)	48.1 (12.3)	54.7 (10.2)
Sex			
Male	171 (69.2%)	120 (52.6%)	93 (80.2%)
Female	76 (30.8%)	108 (47.4%)	23 (19.8%)
Region, *n* (%)			
North	28 (11.3%)	85 (37.3%)	48 (41.4%)
Northeast	83 (33.6%)	17 (7.5%)	49 (42.2%)
Central	106 (42.9%)	105 (46.1%)	11 (9.5%)
Others	30 (12.1%)	21 (9.2%)	8 (6.9%)
Thai ethnicity	242 (98%)	227 (99.6%)	114 (98.3%)
Longest occupation, *n* (%)			
Agriculture	66 (26.7%)	30 (13.2%)	57 (49.1%)
Non‐agriculture	174 (70.4%)	198 (86.8%)	57 (49.1%)
Missing details	7 (2.8%)	0	2 (1.7%)
Hepatitis B, *n* (%)			
Positive	7 (2.8%)	185 (81.1%)	56 (48.3%)
Negative	239 (96.8%)	30 (13.2%)	46 (39.7%)
Missing details	1 (0.4%)	13 (5.7%)	14 (12.1%)
Hepatitis C, *n* (%)			
Positive	1 (0.4%)	16 (7%)	22 (19%)
Negative	239 (96.8%)	203 (89%)	77 (66.4%)
Missing details	7 (2.8%)	9 (3.9%)	17 (14.7%)
Chemical exposure			
Glyphosate (nM mean [min–max])	38.1 (nd–291.50)	120 (nd–4711.90)	40.7 (nd–161.80)
AMPA (nM mean [min–max])	70.0 (nd–167.93)	107 (nd–415.1)	285 (nd–1977.2)
PPA (nM, mean [min–max])	2790 (36.05–18,526)	5340 (19.23–22,641)	4670 (41.22–15,019)

Abbreviations: n, number of samples; nd, Not detected as concentration below limit of detection.

**FIGURE 1:**
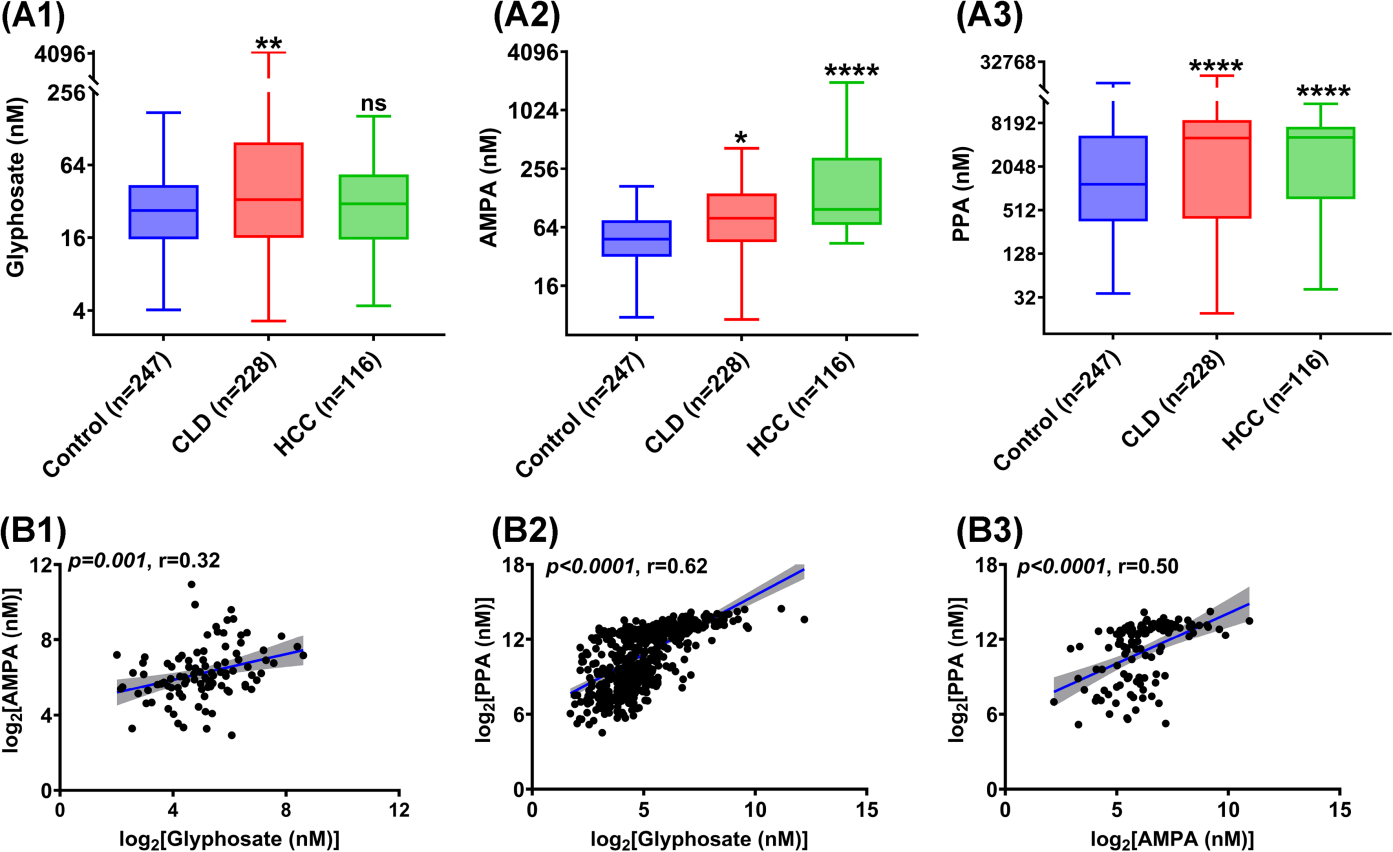
Glyphosate and its metabolites, AMPA and PPA, were assessed in hospital controls, chronic liver disease (CLD) patients, and hepatocellular carcinoma (HCC) cases. (A1) CLD cases showed significantly higher glyphosate levels than hospital controls. (A2–A3) AMPA and PPA levels were significantly elevated in the CLD and HCC groups. (B1–B3) Strong correlations were observed among glyphosate, AMPA, and PPA levels. ‘Control’ refers to hospital controls. A1–A3 panels display the y‐axis on a log2 scale. ‘*n*’ represents sample sizes. Box plots were used, and statistical differences were assessed using the Kruskal–Wallis test.

Higher levels of urinary glyphosate were observed in CLD patients compared to all other groups (Figure 1A1), while the levels in HCC did not significantly differ from hospital controls. The highest level of AMPA was in the HCC group (*p* < .0001; Figure 1A2), and its level in CLD was also higher than in hospital controls (*p* = .02). Levels of PPA (Figure 1A3) were higher in the HCC and CLD groups, relative to hospital controls (*p* < .0001 for each comparison). The urinary levels of the three compounds were highly correlated (Figure 1B1–B3), particularly PPA with glyphosate and AMPA (*r* > 0.50 and *p* < .0001 for both comparison).

The results of the analysis by the odds ratios (OR) for the upper quartiles of exposure in Table 2 demonstrate a significant association between elevated levels of glyphosate, AMPA, and PPA and increased risks of CLD and HCC compared to hospital controls. Glyphosate exposure shows a significant trend for CLD risk, with an OR of 1.47 (*p* = .0004, 95% CI 0.89–2.42) in the upper quartile. AMPA is significantly associated with HCC, with the upper quartile exhibiting an OR of 3.97 (*p* = .0017, 95% CI 1.80–8.75), indicating a strong correlation between elevated AMPA levels and HCC risk. PPA shows the most pronounced associations, with a steady increase in both CLD and HCC risks across quartiles, culminating in an OR of 1.93 for CLD and 3.85 for HCC in the upper quartile (*p* < .0001, 95% CI 1.13–3.12 and *p* = .0003, 95% CI 1.94–7.65, respectively). These findings suggest that elevated concentrations of glyphosate, AMPA, and PPA are significantly linked to increased liver disease risk, further supported by the results of their trend tests, which were statistically significant (except for AMPA and CLD). To see whether comparisons of the upper quartile of exposure to lower levels would yield more stable results, we conducted additional exploratory analyses by reanalyzing the same data, combining quartiles 1–3 as a referent group for quartile 4. Accordingly, we found no statistically significant odds ratios for glyphosate (OR = 0.94 and 1.19 for CLD and HCC, respectively), nor for AMPA and CLD (OR = 1.84). However, for AMPA, there was an elevated odds ratio for HCC (OR = 3.94, 95% CI 1.80–8.65). For PPA, the odds of both CLD (OR = 2.32, 95% CI 1.58–3.40) and HCC (OR = 2.60, 95% CI 1.63–4.14) were elevated.

**TABLE 2:** Odds Ratios for Glyphosate and its metabolites.

	Hospital controls	CLD cases		HCC cases	
	*n* (%)	*n* (%)	OR (95% CI)	*n* (%)	OR (95% CI)
*Glyphosate quartiles*					
Q1 (<LOD–4.0 nM)	70 (39.8%)	70 (39.8%)	1.00 (Reference)	36 (20.5%)	1.00 (Reference)
Q2 (4.1–15.5 nM)	57 (51.8%)	41 (37.3%)	0.72 (0.41–1.24)	12 (10.9%)	0.39 (0.17–0.88)
Q3 (15.6–26.8 nM)	60 (48.0%)	29 (23.2%)	0.48 (0.26–0.86)	36 (28.8%)	1.17 (0.62–2.20)
Q4 (26.9–172.4 nM)	60 (33.3%)	88 (48.9%)	1.47 (0.89–2.42)	32 (17.8%)	1.04 (0.58–1.87)
*p* value for trend			0.0004		0.0348
*AMPA quartiles*					
Q1 (<LOD–7.5 nM)	216 (42.8%)	200 (39.6%)	1.00 (Reference)	89 (17.6%)	1.00 (Reference)
Q2 (7.6–31.9 nM)	10 (58.8%)	5 (29.4%)	0.54 (0.17–1.73)	2 (11.8%)	0.54 (0.11–2.54)
Q3 (32.0–48.4 nM)	10 (45.5%)	5 (22.7%)	0.54 (0.17–1.73)	7 (31.8%)	1.70 (0.63–4.60)
Q4 (48.5–167.7 nM)	11 (23.4%)	18 (38.3%)	1.77 (0.79–3.95)	18 (38.3%)	3.97 (1.80–8.75)
*p* value for trend			0.1803		0.0017
*PPA quartiles*					
Q1 (19.2–36.2 nM)	62 (47.7%)	54 (41.5%)	1.00 (Reference)	14 (10.8%)	1.00 (Reference)
Q2 (36.3–361.9 nM)	61 (53.5%)	31 (27.2%)	0.57 (0.32–1.01)	22 (19.3%)	1.60 (0.75–3.41)
Q3 (362.0–1167 nM)	62 (48.8%)	39 (30.7%)	0.72 (0.42–1.25)	26 (20.5%)	1.86 (0.89–3.89)
Q4 (1168–18,561 nM)	62 (28.2%)	104 (47.3%)	1.93 (1.13–3.12)	54 (24.5%)	3.85 (1.94–7.65)
*p* value for trend			<0.0001		0.0003

Abbreviations: n, number of samples; OR, odds ratio; Trend test: chi‐square test for a linear trend in the odds ratios.

### 3.2 Regional glyphosate usage and HCC risk

Since there are varying types and intensities of agriculture and herbicide usage practiced in the different geographical regions of Thailand,^21^ we stratified the data into five regions (Figure S1A). The northeastern region of Thailand, for example, had the highest rates of pesticide poisonings in the previous 10 years (5.31 cases per 100,000 people), followed by the northern, central, and southern regions (4.57, 2.99, and 0.48 cases per 100,000, respectively).^22^ The study by Pupacdi et al., reported significantly higher herbicide usage in the northern region of Thailand, accounting for 55% of HCC cases and 26% of hospital controls, compared to the whole country (29% and 14%) and the northeast region (5% and 18%). This study also found an increased HCC risk associated with drinking water sources in regions with high glyphosate levels, particularly in the north, where drinking tap or well water (compared to bottled water) posed a greater risk.^18^ These findings led us to investigate regional differences in urinary glyphosate levels and its metabolites.

### 3.3 Elevated urinary glyphosate and liver damage biomarker associations

Our study confirmed these findings by demonstrating significantly elevated urinary levels of glyphosate and its metabolites (AMPA and PPA) in the northern region compared to other (non‐northern) regions (Figure S2). CLD cases with cirrhosis exhibited significantly higher glyphosate levels than non‐cirrhotic cases (Figure S3), indicating a correlation between glyphosate exposure and disease severity. Additionally, glyphosate exposure was positively associated with liver damage biomarkers, including AST/ALT ratio, APRI score, FIB‐4 score, and NAFLD‐FS score (Figure S4).

### 3.4 Regional glyphosate differences

**FIGURE 2:**
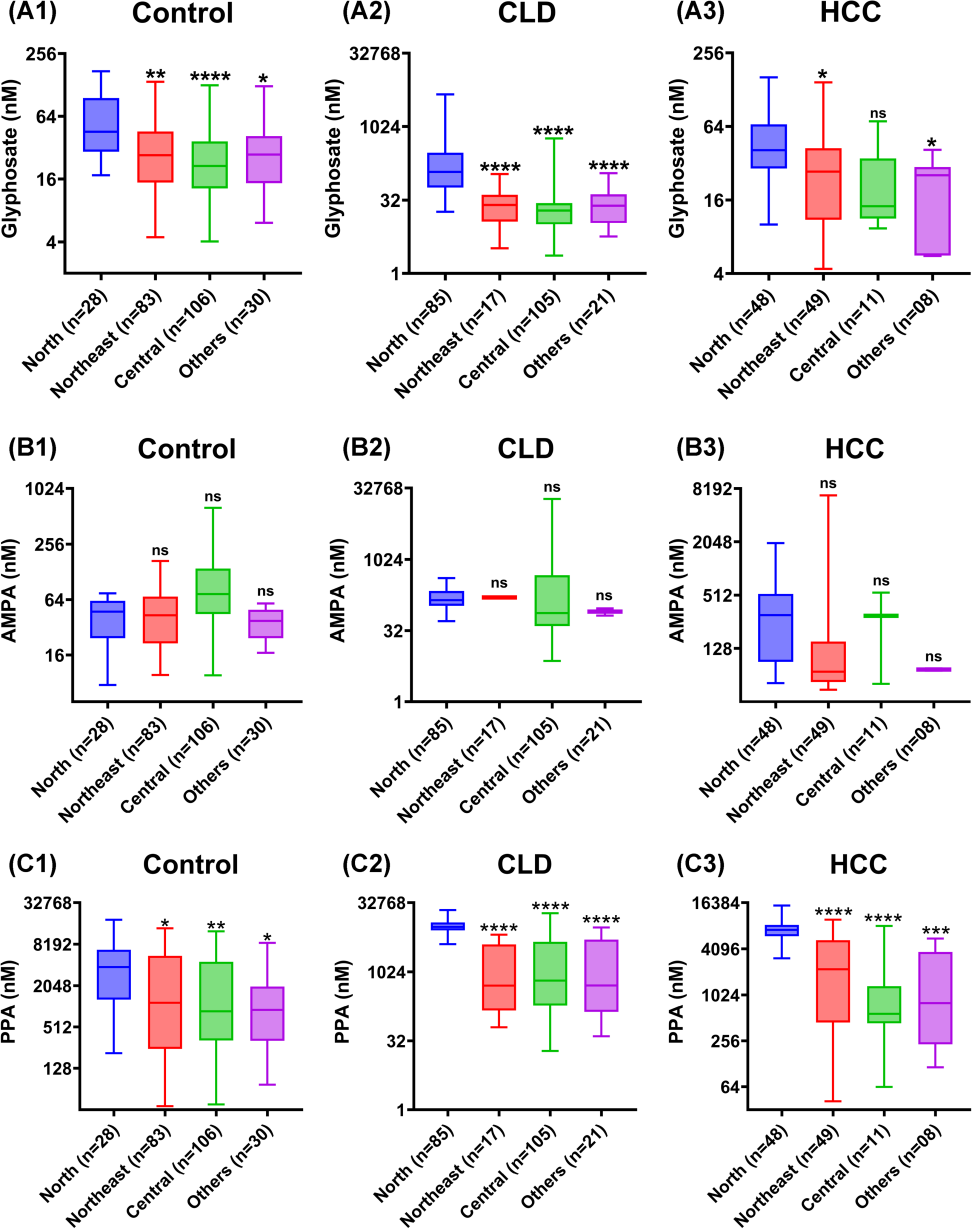
Regional concentrations of glyphosate and its metabolites (AMPA and PPA) at TIGER‐LC clinical centers. Glyphosate levels were highest in the North for hospital controls (A1), CLD (A2), and HCC (A3). AMPA showed no regional differences (B1–B3), while PPA was elevated in the North (C1–C3). “Control” refers to hospital controls. All panels display the y‐axis on a log2 scale. Sample sizes are indicated by “*n*,” and data are presented in box plots with statistical differences assessed using the Kruskal–Wallis test.

Individuals from the northern region consistently showed elevated levels of both glyphosate and PPA in HCC cases, CLD cases, and hospital controls compared to the northeast, central, and other regions (Figure 2 and Tables S1–S5). Specifically, hospital controls in the northern region had a median glyphosate level of 43 nM, compared to 25 nM in the central region and 27 nM in the northeast. This pattern was observed consistently across HCC and CLD cases for all three chemicals. Among participants from the northern region, individuals with CLD exhibited higher levels of glyphosate, AMPA, and PPA compared to those with HCC and the hospital control group (Figure S5). These results emphasize the distinct regional variation in glyphosate exposure, particularly in the northern region, which corresponds to higher HCC risk linked to contaminated dietary and drinking water sources as reported by Pupacdi et al.^18^

### 3.5 Glyphosate and its metabolites in food and water samples

We analyzed convenience food and water samples collected from various marketplaces in Chiang Mai and Sukhothai provinces in the north, and Mahachai and Samutsakorn provinces in the central region in 2020 (Table S6). Glyphosate was detected in crab paste, pork, and fish sauce (23–345 μg/g) but was low to undetectable in tap water and bottled water samples. In the food and water samples from Chiang Mai, we found low levels of glyphosate, AMPA, and PPA in bottled and tap water, but somewhat higher levels in a single well water sample. Fermented fish and crab paste samples were positive for glyphosate and AMPA, with extremely high and variable levels of PPA (Table S6).

### 3.6 Associations by sex, occupation, and HCV/HBV infection

**FIGURE 3:**
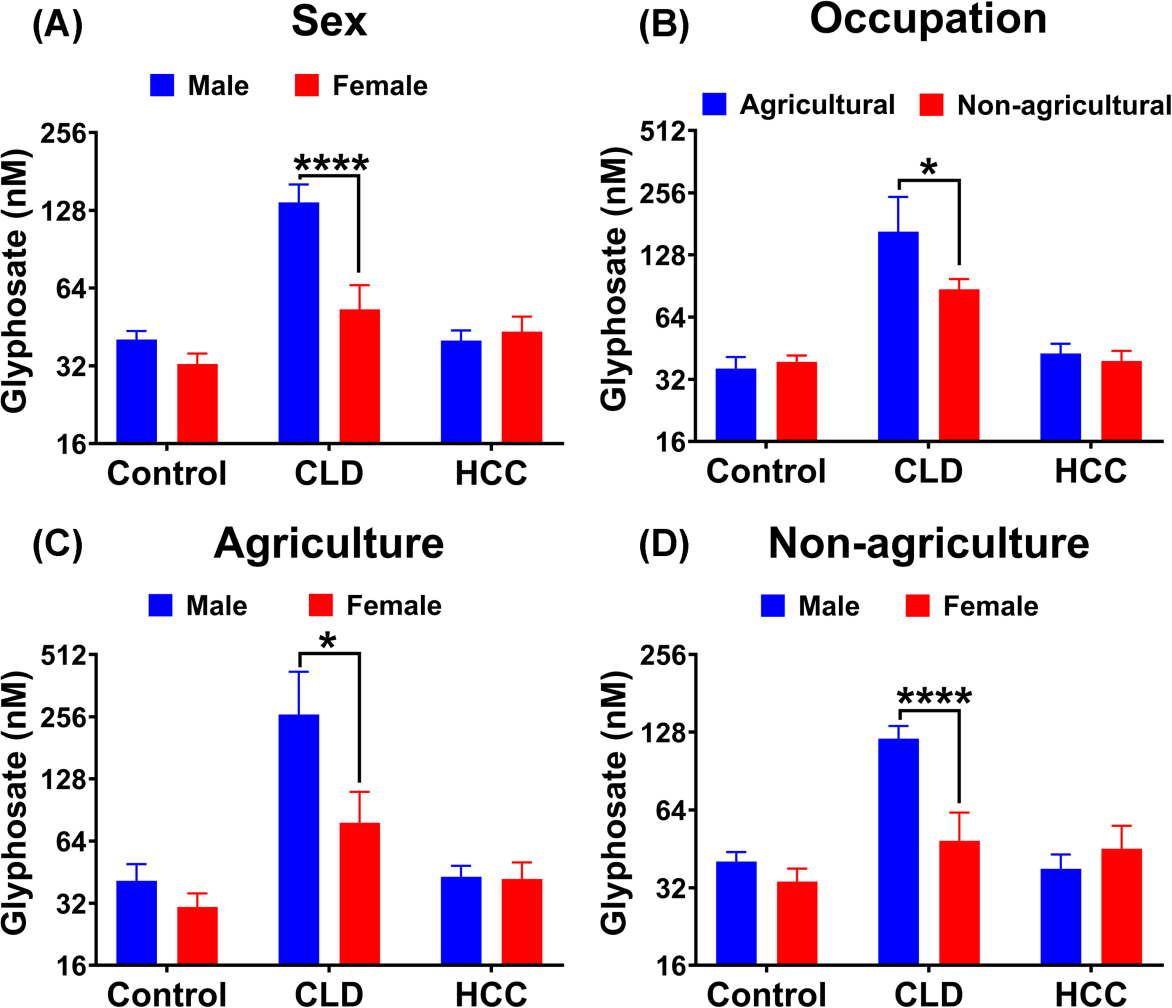
Epidemiological analysis of urinary glyphosate levels at TIGER‐LC centers by sex and occupation. (A) CLD males had higher glyphosate levels than CLD females, with no significant sex differences in hospital controls or HCC. (B) CLD agricultural workers had higher glyphosate levels than non‐agricultural workers, with no significant difference in hospital controls or HCC. (C) Male agricultural workers with CLD had higher glyphosate levels than females, with no significant difference in hospital controls or HCC. (D) Non‐agricultural male workers with CLD had higher glyphosate levels than females, with no significant difference in hospital controls or HCC. “Control” refers to hospital controls. All panels display the y‐axis on a log2 scale. Data were analyzed using two‐way ANOVA; “*n*” denotes the number of samples.

We stratified glyphosate levels by sex (Figure 3A), and by occupation, contrasting those who worked in agriculture (Figure 3B) compared to all non‐agriculture sectors combined. Overall, we found that glyphosate levels were higher in males compared to females in the CLD group, where males had over 2‐fold higher levels of glyphosate compared to their female counterparts (Figure 3A,C,D). Participants with CLD employed in the agricultural sector had higher levels of glyphosate compared to those employed in non‐agriculture sectors (Figure 3B), and the same pattern was observed when only males were analyzed (data not shown). Similar findings were observed for AMPA and PPA in relation to sex and occupation for CLD cases and showed similar trends in the HCC cases (Figure S6). Glyphosate levels were higher in the HCV positive subjects compared to HCV negative persons, but only in the CLD group and were comparable across sexes (Figure S7). HBV infection was inversely associated with glyphosate levels.

### 3.7 Regression‐based models for HCC and CLD risk

In addition to the analyses above, we used logistic regression models to assess the effects of each chemical as a continuous variable, adjusted for gender, agricultural versus non‐agricultural occupation, region, ever versus never drinker, HBV and HCV (positive vs. negative for each virus) and consumption of raw or fermented fish (Tables S7 and S8). Compared to hospital controls, the risk of CLD increased in a linear fashion with increasing levels of glyphosate, and with increasing levels of AMPA and PPA (*p* <.0001 for each compound; Table S7). But for HCC the regression coefficients for each of these exposures failed to reach statistical significance (*p* = .065, 0.41, and 0.28 for glyphosate, AMPA, PPA, respectively; Table S8). Similar results were observed when we dichotomized each compound at the median values of their distributions in the hospital control group (data not shown).

**FIGURE 4:**
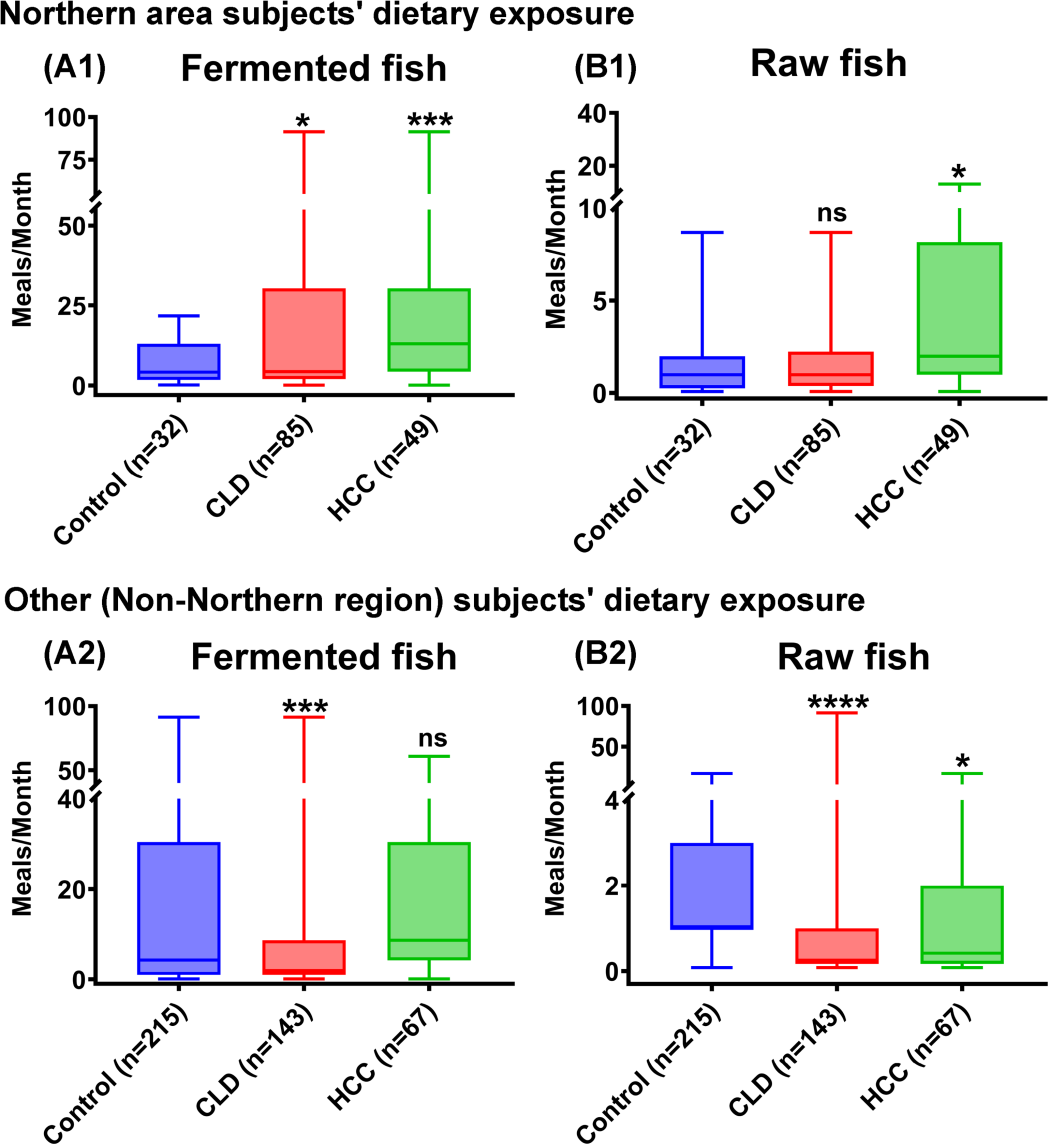
Dietary glyphosate exposure through fish consumption was studied in Northern Thailand. (A1) CLD and HCC cases consumed more fermented fish monthly than hospital controls. (B1) HCC cases consumed more raw fish than hospital controls. In non‐Northern regions, (A2) CLD cases consumed fewer fermented fish and (B2) fewer raw fish compared to controls. “Control” refers to hospital controls. Box plots are shown, with statistical differences assessed using the Welch *t*‐test. “*n*” refers to the sample sizes.

Using questionnaire data, we also identified any regularly consumed food items that were differentially distributed between the case and hospital control groups. More frequent consumption of fermented fish was reported in the northern region by HCC and CLD cases compared to hospital controls (Figure 4A1, A2), but we did not observe this disparity in the other regions. Data from the northern region also revealed a higher frequency of raw fish consumption in HCC compared to the other groups (Figure 4B1, B2).

## 4 DISCUSSION

Herbicide use over the recent decades to protect crops from weed competition and maximize yields has increased nearly fourfold in Thailand, one of the world's top food exporters.^21^ Glyphosate accounts for 40%–50% of agricultural herbicide use in Thailand, followed by paraquat dichloride (15%–20%) and 2,4‐d dimethyl ammonium (11%–18%).^23^ Glyphosate has a relatively short environmental half‐life of approximately 47 days, due to its rapid breakdown by soil microbes, as reported by the Weed Science Society of America (WSSA) Herbicide Handbook.^24^ However, trace concentrations of glyphosate were detected in the roots and plant tissues of Canadian forests for up to 12 years, indicating its persistence in certain environments over long periods.^25^ Numerous studies have shown that these glyphosate residues are detectable in soil, surface water, groundwater, food, and human biospecimens.^21^, ^26^, ^27^ Glyphosate and its metabolites were found in 97% of biological samples from farmers, and in 98% of non‐farmer samples in a recent study, consistent with the use of this chemical in both residentials yards and farmlands, and suggesting that food and water contamination, possibly due to farmland water runoff, may contribute to overall population exposures.^28^

In this study, glyphosate and its metabolites were present in the urine samples of HCC and CLD cases and hospital controls, as well as in water and food samples from different regions of the country. Higher exposure levels were observed in the northern region of Thailand than elsewhere, where there is significantly greater use of the herbicide due to the types of crops grown there. Glyphosate is typically applied by farmers with backpack sprayers, machine sprayers, and by hand.^29^ Many fail to wear adequate protective clothing and equipment to prevent exposure. Questionnaire data from the parent TIGER‐LC study (Pupacdi et al.) indicated that self‐reported herbicide usage was much more prevalent among farmers in the northern region of Thailand (55% of HCC cases) compared to the country as a whole (29%). Sex, occupation, and chronic HCV infection were also associated glyphosate levels in that study.^18^ In the case of HCV, glyphosate levels were higher in HCV‐positive CLD patients, compared to HCV‐negative CLD patients. This was not unexpected, as chronic infection with HCV leads to cirrhosis, which impairs the liver's ability to metabolize toxicants such as glyphosate, resulting in the accumulation of the parent compound, as reflected in the urine measurements.^30^, ^31^ However, it is crucial to consider the possibility of reverse causation, where slowed metabolism in CLD patients—particularly those with HCV infection—may cause elevated glyphosate concentrations, rather than glyphosate directly contributing to liver disease progression.^30^ This potential bias could affect the interpretation of our results, especially given the absence of specific HCV‐RNA serum titer data for this study, as low viral loads do not necessarily prevent cirrhosis progression.^32^ Therefore, further research is needed to clarify the directionality of this association. In the case of HBV, we do not have an explanation for our observation of lower urinary glyphosate levels in persons with chronic HBV compared to HBV‐negative patients. HBV does not often lead to cirrhosis, unlike HCV, and so there must be other mechanisms underlying the latter observation.

Glyphosate's potential adverse hepatic effects, including its ability to inhibit liver mitochondrial oxidative phosphorylation, were first reported in the early 1980s. Glyphosate can act as a protonophore, increasing the permeability of the mitochondrial membrane to protons and Ca^2+^, as well as promote the generation of reactive oxygen species, resulting in oxidative stress.^33^ Studies show that after chronic exposure to glyphosate, oxidative stress indicators were elevated in the rat liver and kidney, using a maximum glyphosate exposure dosage of 7 mg/L in drinking water.^9^ In rats fed 4.87 mg/kg body weight glyphosate every 2 days for 75 days, hepatic histological alterations and clinical biochemical changes were observed.^34^ Additionally, rodents chronically fed low doses of glyphosate develop liver adenoma and exhibit signs of hepatotoxicity, liver congestion, necrosis, and DNA damage of liver cells consistent with NAFLD and its progression to NASH. Several studies show that, in rodent livers, glyphosate inhibits fatty acid oxidation and increases fat and cholesteryl ester levels, resulting in increased lipid mass per gram of liver.^5^, ^35^ Consistent with such findings, we observed associations between glyphosate and cirrhosis in the CLD cases, and with their clinical biomarkers of liver steatohepatitis. Taken together, such evidence may suggest that the higher levels of glyphosate and its metabolites in CLD compared to HCC and hospital controls reflect impaired metabolic capacity associated with chronic HBV, HCV, or alcoholic liver disease. This mechanism is specific to CLD cases without liver cancer. In liver cancer cases, the pathological processes are more complex, and the exact mechanisms by which glyphosate may contribute to liver toxicity require further investigation, as they likely involve additional factors beyond impaired metabolic capacity due to chronic viral or alcoholic hepatitis.

Previous studies described how glyphosate and AMPA can also induce DNA single and double strand breaks and caused purine and pyrimidine oxidation in human peripheral blood mononuclear cells (PBMCs).^36^ Mills et al., not only showed similar exposure findings in an agriculturally exposed population in the USA, but reported a dose‐dependent glyphosate association in NASH development, which leads to liver fibrosis and increases the incidence of HCC development.^3^ This supports our observation of the correlations between FIB score and glyphosate exposure.^37^

Exposure to glyphosate metabolites, AMPA and PPA, presents potential human health hazards, and urinary pesticide exposure assessment in liver disease is crucial for providing accurate, non‐invasive measurements that link pesticide burden to liver cancer risk.^10^ Manas et al., reported AMPA, a primary product of glyphosate oxidation, as a potential genotoxicant.^38^ AMPA has a greater environmental persistence than glyphosate, with 76–240 days and 2–97 days of soil half‐life, respectively, and there is evidence indicating that AMPA may also pose a risk for groundwater pollution.^39^, ^40^ In humans, most of the ingested or inhaled glyphosate dose remains unchanged from the point of exposure to excretion in urine and feces, with a small amount metabolized to AMPA (ATSDR 2020).^41^ In the environment, glyphosate can remain in the water and soil, where bacteria metabolize the parent compound to AMPA. Although AMPA was quantifiable in only 20% of our urine samples, which could be due to its very low concentrations, and unknown AMPA stability in urine, these results are compatible with prior reports.^8^, ^42^ For this investigation, a single time‐point collected sample from each patient, taken between 2011 and 2016 and stored at −70°C, was used for analyte measurement. A positive measurement in a urinary sample typically reflects recent exposure within the past few weeks to months, depending on the compound's metabolism and excretion rate. Another study showed detectable analyte measurements in urine from agricultural workers up to 7 days after glyphosate exposure.^43^ In that study, farmers with high lifetime exposure had higher analyte levels, while controls with minimal lifetime exposure had lower levels. These findings suggest that while short‐term exposure is positively measured, high cumulative exposure results in higher baseline metabolite levels.^8^, ^43^ Glyphosate and its metabolites, AMPA and PPA, are generally stable in urine for several months at low temperatures like −20°C or −80°C.^44^, ^45^, ^46^, ^47^, ^48^ However, their long‐term stability is not well‐documented and needs further investigation. This represents a limitation in our study, as it may affect the interpretation of chronic exposure levels. More investigation is needed to fully understand the stability and degradation of these metabolites over extended periods to accurately assess chronic exposure levels. In contrast to AMPA which resulted from the oxidation of the parent compound, PPA forms as a result of the catabolism of glyphosate, and has multiple sources in the environment, including other phosphorus‐based pesticides.^44^, ^48^, ^49^

Furthermore, we acknowledge the critical role of timing of exposure in accurately assessing exposure‐outcome associations. Our study captured only the most recent exposures among participants, given the short half‐lives of these chemicals, as described above. Urinary levels would thus represent integrated occupational, dermal, inhalation, and dietary routes of exposure over a very short time frame in relation to the decades‐long lag time to develop CLD and HCC. Therefore, chronic exposure history could also influence outcomes. In that context, the advantage of including occupation in the analysis is that is represents a long history of agricultural exposure to glyphosate in the vast majority of farmers, but it does not directly measure chronicity of exposure. Similarly, people may have lived in the same region and consumed the same source of drinking water and ate the same foods over their lifetimes, further contributing to the exposure history of glyphosate and its metabolites.

As glyphosate exposure was observed for individuals in both agricultural and non‐agricultural occupations, it is possible that persons in our study were exposed via contaminated food and water. We found higher consumption of raw and fermented fish in HCC cases compared to hospital controls. Fermented freshwater fish, called *pla‐ra*, and crab paste are widely used flavoring agents in Thai meals.^50^ Convenience samples of crab paste and fermented fish from different marketplaces in the north and central regions of Thailand were found to have significant glyphosate contamination, and a positive association between HCC and CLD cases and self‐reported frequency of fermented fish consumption was observed. These findings support our hypothesis that glyphosate exposure in humans can occur via contaminated food intake and is not exclusive to agricultural occupations. We further recognize that indicators of socio‐economic status, such as income and education, may also impact exposure levels, and need to be taken into account in further work on this topic. In our previous article on this study population (Pupacdi et al.), low educational attainment was strongly associated with both CLD and HCC.^18^

This hospital‐based case control study has notable strengths, including the relatively large sample size, standardized recruitment and data and sample acquisition from participants, and rigorous methods of bioassay. But the case–control design may have introduced recall bias impacting the reporting of potentially important covariates such as lifestyle exposures and food consumption history. Prospective studies with baseline assessment of such factors and follow‐up for disease progression in persons with early stage CLD would be helpful to sort this out. Results are not necessarily generalizable to other populations exposed to glyphosate, for example, through foods like crab paste that are highly specific to areas of Thailand, but they may be relevant to populations where intense use of the chemical in agriculture has led to concerns about contamination of drinking water and other routes of exposure to the non‐agricultural sector as well. Nevertheless, the results of this study warrant future research, including detailed and systematic investigations of food products such as raw and fermented fish, and quantitative determinations of the half‐life and stability of glyphosate, AMPA, and PPA in body fluids such as urine and blood. Future experimentation should also investigate the effects of these compounds on DNA integrity in human hepatocytes in vitro.

## 5 CONCLUSION

This study identifies glyphosate exposure as a potential environmental factor associated with chronic liver disease and HCC. If confirmed in future investigations, these associations draw further attention to the need for glyphosate exposure regulations and controls to reduce its burdens on human health.

## AUTHOR CONTRIBUTIONS

**Daxesh P. Patel:** Conceptualization; data curation; formal analysis; investigation; methodology; software; validation; visualization; writing – original draft. **Christopher A. Loffredo:** Conceptualization; data curation; formal analysis; investigation; software; validation; visualization; writing – original draft. **Benjarath Pupacdi:** Data curation; formal analysis; investigation; resources; validation; writing – original draft. **Siritida Rabibhadana:** Data curation; resources; validation; writing – review and editing. **Panida Navasumrit:** Data curation; resources; validation; writing – review and editing. **Jittiporn Chaisaingmongkol:** Data curation; resources; validation; writing – review and editing. **Leila Toulabi:** Writing – review and editing. **Majda Haznadar:** Writing – review and editing. **Bhavik Dalal:** Writing – review and editing. **Mohammed Khan:** Writing – review and editing. **Joshua Stone:** Writing – review and editing. **Vajarabhongsa Bhudhisawasdi:** Data curation; resources; validation; writing – review and editing. **Nirush Lertprasertsuke:** Data curation; resources; validation; writing – review and editing. **Anon Chotirosniramit:** Data curation; resources; validation; writing – review and editing. **Chawalit Pairojkul:** Data curation; resources; validation; writing – review and editing. **Chirayu U. Auewarakul:** Data curation; resources; validation; writing – review and editing. **Thaniya Sricharunrat:** Data curation; resources; validation; writing – review and editing. **Kannika Phornphutkul:** Data curation; resources; validation; writing – review and editing. **Suleeporn Sangrajrang:** Data curation; resources; validation; writing – review and editing. **Anuradha Budhu:** Data curation; resources; validation; writing – review and editing. **Chulabhorn Mahidol:** Data curation; resources; validation; writing – review and editing. **Xin W. Wang:** Data curation; resources; supervision; writing – review and editing. **Frank J. Gonzalez:** Data curation; resources; writing – review and editing. **Mathuros Ruchirawat:** Data curation; resources; supervision; writing – review and editing. **Curtis C. Harris:** Conceptualization; data curation; funding acquisition; investigation; project administration; supervision; visualization; writing – review and editing.

## FUNDING INFORMATION

Funding was provided in part by the intramural research program of the Center for Cancer Research, National Cancer Institute of the United States (Z01BC010313). Work performed in Thailand, including patient recruitment, data collection, and biospecimen banking, was supported by the Chulabhorn Research Institute, Bangkok, Thailand, and in part by Thailand Science Research and Innovation (TSRI), Chulabhorn Research Institute (Grant no. 49890/4759784).

## CONFLICT OF INTEREST STATEMENT

The authors declare no conflicts of interest.

## ETHICS S TATEMENT

Ethical approval was obtained from the institutional review boards of all participating centers, and all participants provided written informed consent.

## Supporting information


**DATA S1.** Supporting information.

## Data Availability

The dataset underlying this article will be shared upon reasonable request to the corresponding author.
