# Supplementary material for: Associations of chronic liver disease and liver cancer with glyphosate and its metabolites in Thailand
Source: Int J Cancer. 2024 Dec 9;156(10):1885–97. doi: 10.1002/ijc.35282 (PMC11924304; doi:10.1002/ijc.35282)
Supplement: Supplementary file 1 — DATA S1. Supporting information. [file IJC-156-1885-s001.pdf]

Supplementary Materials and Methods

Associations of Chronic Liver Disease and Liver Cancer with Glyphosate and its Metabolites in Thailand

Daxesh P. Patel, Christopher A. Loffredo, Benjarath Pupacdi, Siritida Rabibhadana, Panida Navasumrit, Jittiporn Chaisaingmongkol, Leila Toulabi, Majda Haznadar, Bhavik Dalal, Mohammed Khan, Joshua Stone, Vajarabhongsa Bhudhisawasdi, Nirush Lertprasertsuke, Anon Chotirosniramit, Chawalit Pairojkul, Chirayu U. Auewarakul, Thaniya Sricharunrat, Kannika Phornphutkul, Suleeporn Sangrajrang, Anuradha Budhu, Chulabhorn Mahidol, Xin W. Wang, Frank J. Gonzalez, Mathuros Ruchirawat, and Curtis C. Harris, on behalf of the TIGER-LC Consortium

Table of Contents:

Figure S1: Study sites and glyphosate metabolism………………………………………………………. 2

Figure S2: Regional comparison of glyphosate, AMPA, and PPA levels Across TIGER-LC centers …….. 3

Figure S3: Glyphosate and metabolites in cirrhotic compared to non-cirrhotic CLD patients …………….. 4

Figure S4: Pearson's correlation of glyphosate exposure with biochemical markers for CLD patients …… 5

Figure S5: Distribution of glyphosate, AMPA, and PPA in the Northern region for hospital controls and CLD and HCC cases ……………………………………………………………………………... 6

Figure S6: Analysis of AMPA and PPA exposure by occupation …………………………………………. 7

Figure S7: Epidemiological assessment of glyphosate exposure by HBV/HCV status ………………….... 8

Table S1. Characteristics of participants from Chiang-Mai Clinical Center of TIGER-LC………..……… 9

Table S2. Characteristics of participants from NCI-Thailand Clinical Center of TIGER-LC …..………..... 10

Table S3. Characteristics of participants from Srinakarind Clinical Center of TIGER-LC ……………….. 11

Table S4. Characteristics of participants from Chulabhorn Clinical Center of TIGER-LC ……………..... 12

Table S5. Characteristics of participants from Roi Et Clinical Center of TIGER-LC …………………….. 13

Table S6. Glyphosate and its metabolites – AMPA and PPA levels in food and water samples in northern and central provinces ……………………………………………..………………………………... 14

Table S7. Regression model for CLD group compared to Hospital controls …………………………..….. 15

Table S8. Regression model for HCC group compared to Hospital controls …………………………….....16

Figure S1: Study sites and glyphosate metabolism. (A) Map showing locations of the participating Thailand hospitals: Maharaj Nakorn Chiang Mai, Srinagarind, Roi-Et, Chulabhorn Research Institute, and the National Cancer Institute. (B) Glyphosate metabolism: AMPA forms via oxidative cleavage by GOX, rapidly degrading into PPA.

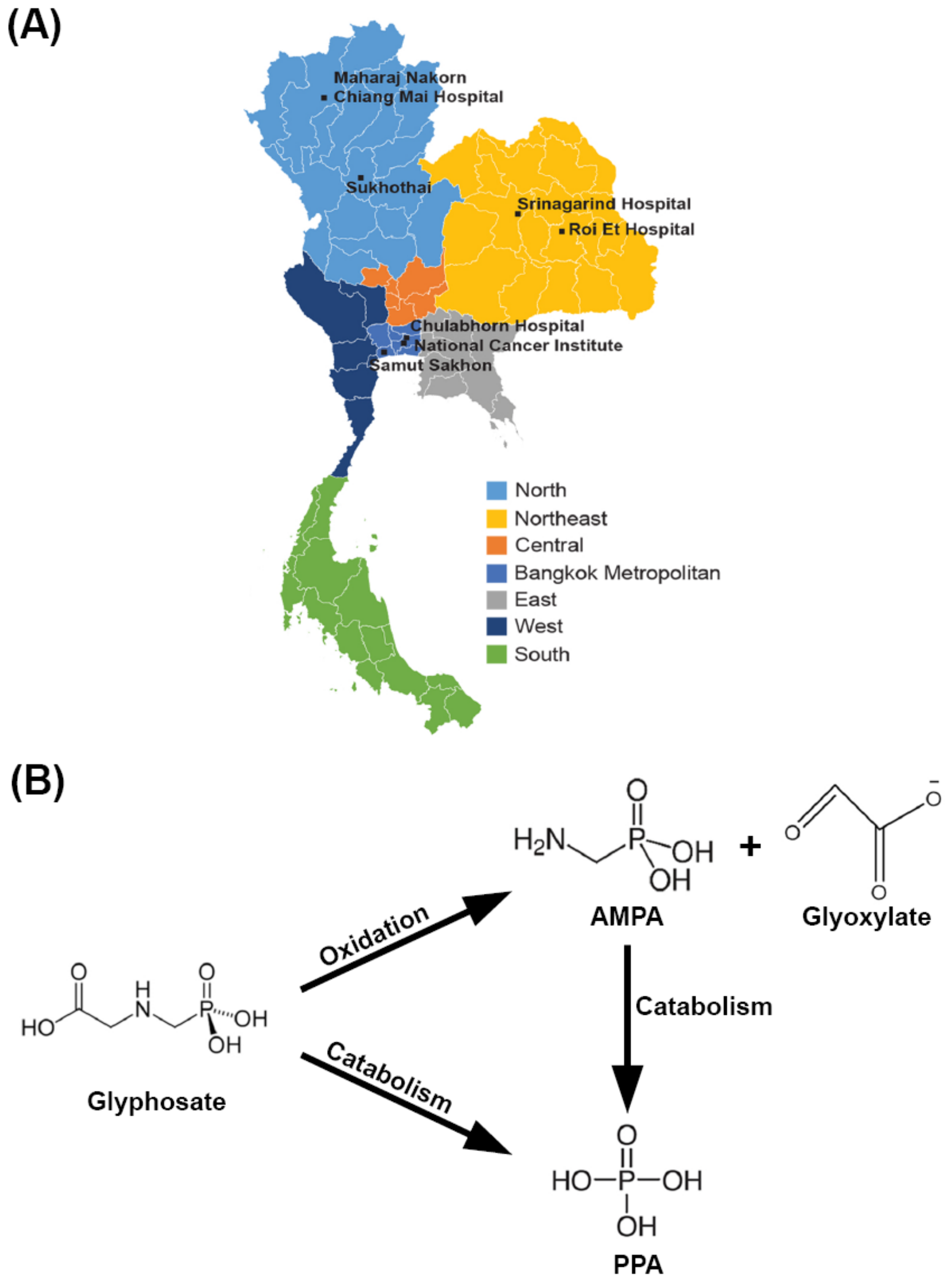

Figure S2: Regional comparison of glyphosate, AMPA, and PPA levels Across TIGER-LC centers. Comparison of glyphosate and its metabolites, AMPA and PPA, between the northern (Chiang Mai) and other (non-northern) regions at TIGER-LC centers (KU, RE, NC, CC). Glyphosate levels were significantly higher in the Northern region for hospital controls (A1), CLD (A2), and HCC cases (A3). AMPA levels were not significantly different in hospital controls (B1) but were elevated in CLD (B2) and HCC (B3) cases in the Northern region. PPA levels were significantly higher in the Northern region across all groups: hospital controls (C1), CLD (C2), and HCC (C3). "Control" refers to hospital controls. All panels display the y-axis on a log2 scale. "n" denotes the number of samples. Data are shown as box plots, with statistical differences determined using the Mann-Whitney test.

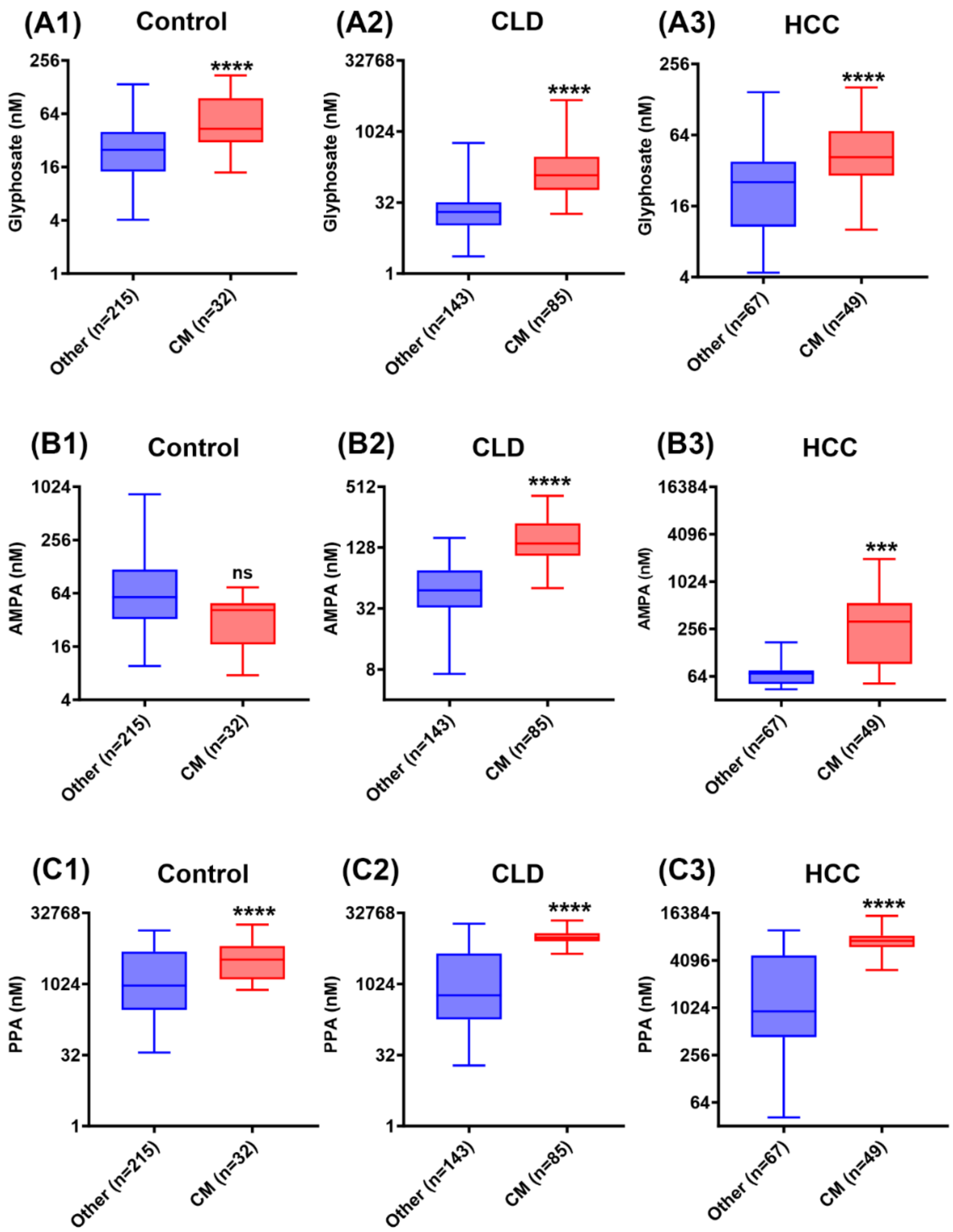

Figure S3: Glyphosate and metabolites in cirrhotic compared to non-cirrhotic CLD patients. Glyphosate exposure was significantly higher in cirrhotic CLD patients compared to non-cirrhotic CLD patients and hospital controls (A). No significant differences were observed between non-cirrhotic CLD patients and controls. PPA (C) and AMPA (B) showed similar trends. 'Control' refers to hospital controls. All panels display the y-axis on a log2 scale. 'n' denotes the total number of samples.

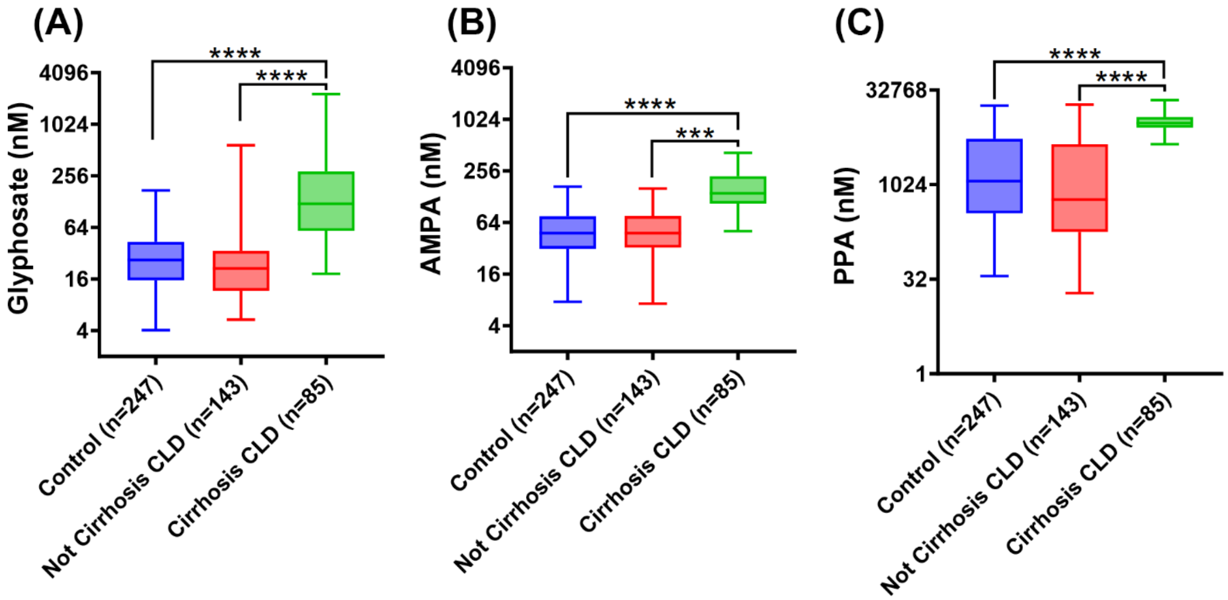

Figure S4: Pearson's correlation of glyphosate exposure with biochemical markers for CLD patients. (A) AST/ALT ratio, (B) APRI, (C) FIB-4, and (D) NAFLD-FS.

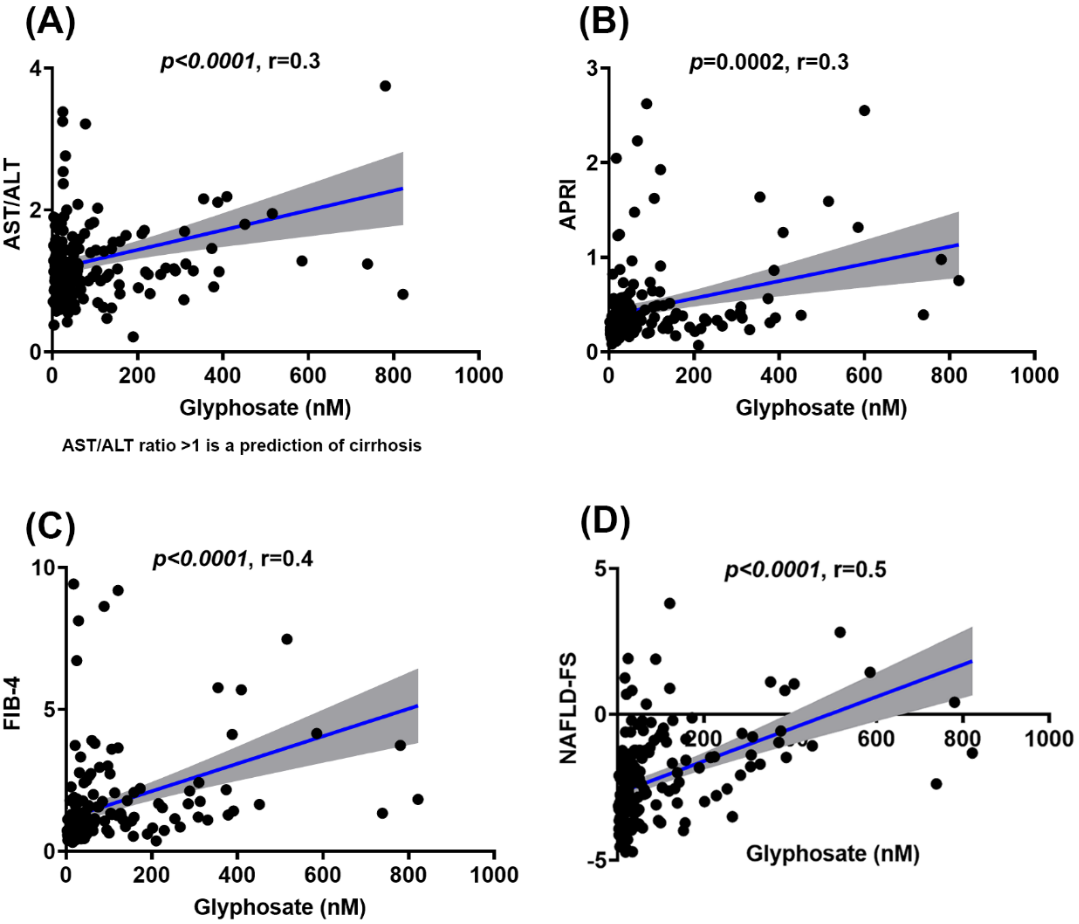

Figure S5: Distribution of glyphosate, AMPA, and PPA in the Northern region for hospital controls and CLD and HCC cases. (A) Glyphosate levels were significantly higher in CLD cases than in hospital controls. (B-C) AMPA and PPA levels were significantly elevated in both CLD and HCC cases compared to controls. "Control" refers to hospital controls. All panels display the y-axis on a log2 scale. Data are shown in box plots; statistical differences were determined using the Kruskal-Wallis test.

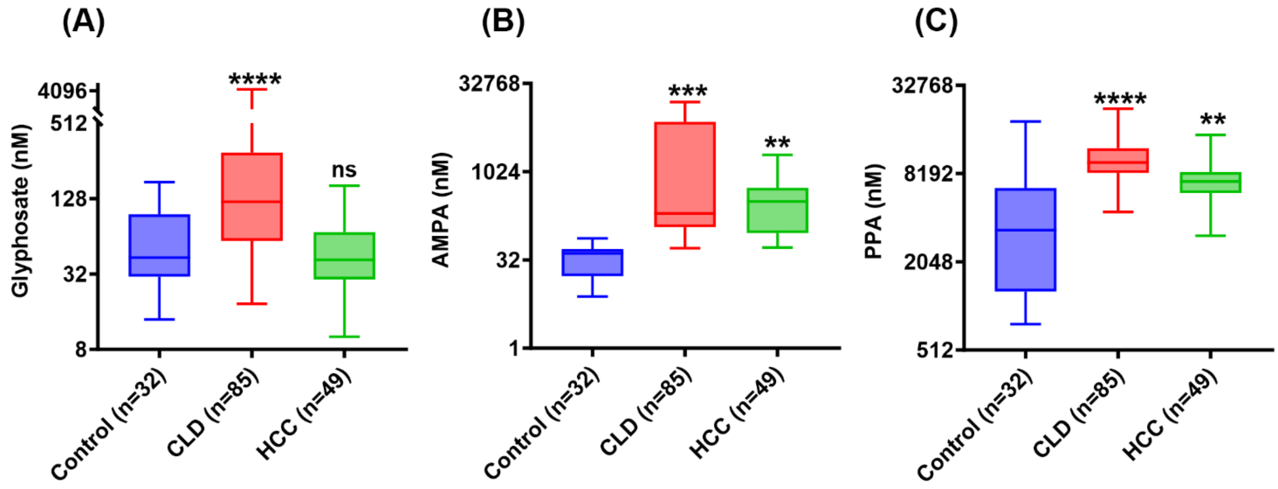

Figure S6: Analysis of AMPA and PPA exposure by occupation. (A) In CLD cases, AMPA levels were significantly higher in agricultural workers than in non-agricultural workers, with no difference in hospital controls or HCC. (B) In HCC cases, agricultural workers had higher PPA levels than non-agricultural workers, with no difference in hospital controls or CLD cases. "Control" refers to hospital controls. All panels display the y-axis on a log2 scale. Statistical differences were determined using two-tailed t-tests, and data are represented with bar plots.

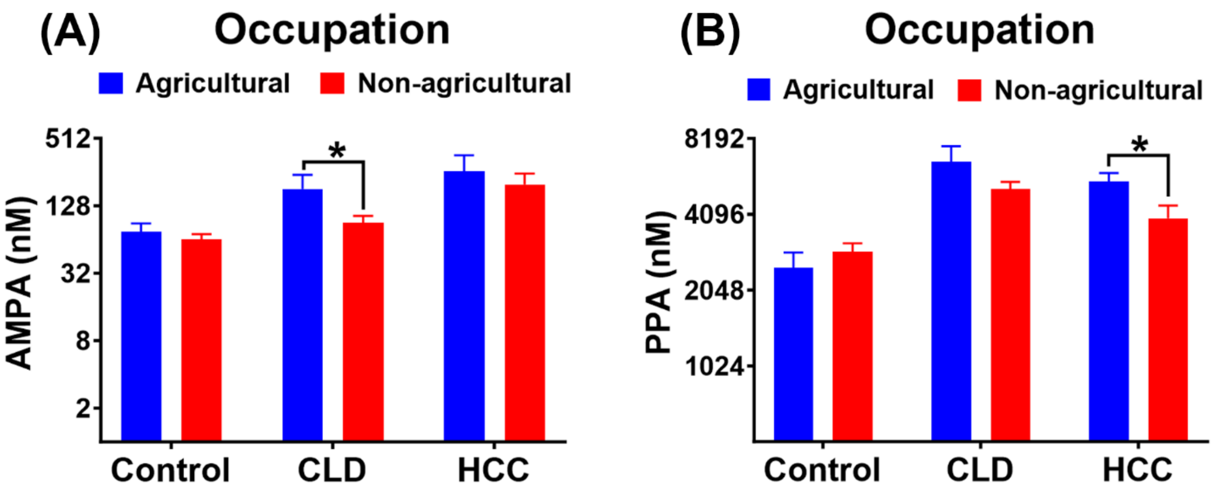

Figure S7: Epidemiological assessment of glyphosate exposure by HBV/HCV status. (A) CLD subjects with HBV- had higher glyphosate levels than HBV+. No significant differences were seen in controls or HCC. (B) CLD subjects with HCV+ had higher glyphosate levels than HCV-. No significant differences were observed in controls or HCC. (C) Higher glyphosate levels in CLD with HBV-/HCV+ and HBV-/HCV- than HBV+/HCV-. (D) Higher glyphosate in CLD males and females with HBV- than HBV+. (E) Higher glyphosate in CLD males and females with HCV+ than HCV-. "Control" refers to hospital controls. All panels display the y-axis on a log2 scale. Statistical differences were determined using two-tailed t-tests. Data is shown with bar plots.

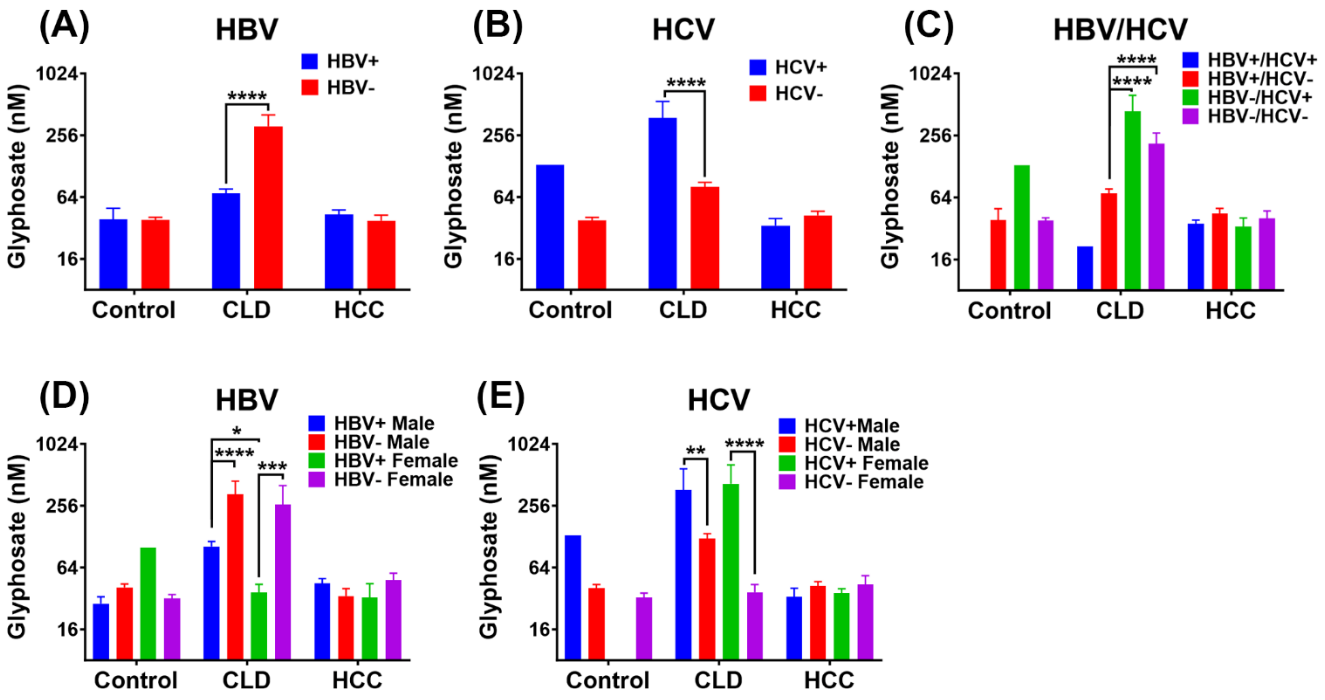

Table S1. Characteristics of participants from Chiang-Mai Clinical Center of TIGER-LC

Chiang-Mai Clinical Center	Hospital Controls	CLD Cases	HCC Cases
**Total No of samples**	32	85	49
**Mean age (SD)**	44.0 (7.5)	55.8 (8.4)	53.1 (9.0)
**Sex**			
**Male**	28 (87.5%)	73 (85.9%)	42 (85.7%)
**Female**	4 (12.5%)	12 (14.1%)	7 (14.3%)
**Hepatitis B, n (%)**			
**Positive**	4 (12.5%)	62 (72.9%)	30 (61.2%)
**Negative**	28 (87.5%)	23 (27.1%)	19 (38.8%)
**Missing details**	0	0	0
**Hepatitis C, n (%)**			
**Positive**	1 (3.1%)	10 (11.8%)	13 (26.5%)
**Negative**	31 (96.9%)	75 (88.2%)	36 (73.5%)
**Missing details**	0	0	0
**Chemical exposure**			
**Glyphosate (nM Median (min-max))**	43.3 (nd-291.5)	121 (nd-4712)	42.0 (nd-161.8)
**AMPA (nM Median (min-max))**	62.1 (nd-141.3)	140 (nd-415.1)	317.6 (nd-1977)
**PPA (nM, Median (min-max))**	3480 (768.0-18526)	9750.0 (4484-22641)	7230 (3080-15019)

SD: Standard deviation; n: Number of samples; nd: Not detected as concentration below limit of detection.

Table S2. Characteristics of participants from NCI-Thailand Clinical Center of TIGER-LC

NCI-Thailand Clinical Center	Hospital Controls	CLD Cases	HCC Cases
**Total No of samples**	148	29	1
**Mean age (SD)**	54.0 (9.0)	55.9 (9.5)	57
**Sex**			
**Male**	109 (73.6%)	13 (44.8%)	1 (100%)
**Female**	39 (26.4%)	16 (55.2%)	0
**Hepatitis B, n (%)**			
**Positive**	3 (2%)	4 (13.8%)	1 (100%)
**Negative**	144 (97.3%)	23 (79.3%)	0
**Missing details**	1 (0.7%)	2 (6.9%)	0
**Hepatitis C, n (%)**			
**Positive**	0	4 (13.8%)	0
**Negative**	144 (97.3%)	23 (79.3%)	1 (100%)
**Missing details**	4 (2.7%)	2 (%)	0
**Chemical exposure**			
**Glyphosate (nM Median (min-max))**	24.6 (nd-127.5)	31.1 (nd-584.9)	21.2
**AMPA (nM Median (min-max))**	57.9 (nd-167.9)	44.4 (nd-78.7)	nd
**PPA (nM, Median (min-max))**	819.0 (38.2-12566)	2410 (40.3-19310)	432.9

SD: Standard deviation; n: Number of samples; nd: Not detected as concentration below limit of detection.

Table S3. Characteristics of participants from Srinakarind Clinical Center of TIGER-LC

Srinakarind Clinical Center	Hospital Controls	CLD Cases	HCC Cases
**Total No of samples**	67	9	44
**Mean age (SD)**	57.0 (9.1)	53.1 (10.4)	54.2 (9.7)
**Sex**			
**Male**	34 (50.7%)	6 (66.7%)	34 (77.3%)
**Female**	33 (49.3%)	3 (33.3%)	10 (22.7%)
**Hepatitis B, n (%)**			
**Positive**	0	1 (11.1%)	10 (22.7%)
**Negative**	67 (100%)	0	24 (54.5%)
**Missing details**	0	8 (88.9%)	10 (22.7%)
**Hepatitis C, n (%)**			
**Positive**	0	1 (11.1%)	7 (15.9%)
**Negative**	64 (95.5%)	1 (11.1%)	27 (61.4%)
**Missing details**	3 (4.5%)	7 (77.8%)	10 (22.7%)
**Chemical exposure**			
**Glyphosate (nM Median (min-max))**	26.9 (nd-212.8)	18.2 (9.7-109.0)	27.4 (nd-147.3)
**AMPA (nM Median (min-max))**	56.8 (nd-151.0)	nd	62.7 (nd-173.0)
**PPA (nM, Median (min-max))**	1080 (36.1-13918)	478 (81.0-6584)	1190 (41.2-9844)

SD: Standard deviation; n: Number of samples; nd: Not detected as concentration below limit of detection.

Table S4. Characteristics of participants from Chulabhorn Clinical Center of TIGER-LC

Chulabhorn Clinical Center	Hospital Controls	CLD Cases	HCC Cases
**Total No of samples**	NA	105	15
**Mean age (SD)**	NA	39.6 (9.8)	57.5 (14.6)
**Sex**	NA		
**Male**		28 (26.7%)	11 (73.3%)
**Female**		77 (73.3%)	4 (26.7%)
**Hepatitis B, n (%)**	NA		
**Positive**		102 (97.1%)	10 (66.7%)
**Negative**		0	1 (6.7%)
**Missing details**		3 (2.9%)	4 (26.7%)
**Hepatitis C, n (%)**	NA		
**Positive**		1 (1%)	1 (6.7%)
**Negative**		104 (99%)	8 (53.3%)
**Missing details**		0 (0%)	6 (40%)
**Chemical exposure**			
**Glyphosate (nM Median (min-max))**	NA	17.3 (nd-266.0)	11.7 (nd-41.2)
**AMPA (nM Median (min-max))**	NA	48.3 (nd-159.4)	73.9 (nd-76.2)
**PPA (nM, Median (min-max))**	NA	559 (19.2-11813)	576 (70.3-6598)

SD: Standard deviation; NA: Samples not available; n: Number of samples; nd: Not detected as concentration below limit of detection.

Table S5. Characteristics of participants from Roi Et Clinical Center of TIGER-LC

Roi Et Clinical Center	Hospital Controls	CLD Cases	HCC Cases
**Total No of samples**	NA	NA	7
**Mean age (SD)**	NA	NA	62.9 (5.8)
**Sex**	NA	NA	
**Male**			5 (71.4%)
**Female**			2 (28.6%)
**Hepatitis B, n (%)**	NA	NA	
**Positive**			5 (71.4%)
**Negative**			2 (28.6%)
**Missing details**			0 (0%)
**Hepatitis C, n (%)**	NA	NA	
**Positive**			1 (14.3%)
**Negative**			5 (71.4%)
**Missing details**			1 (14.3%)
**Chemical exposure**			
**Glyphosate (nM Median (min-max))**	NA	NA	21.1 (6.0-53.9)
**AMPA (nM Median (min-max))**	NA	NA	75.9 (nd-75.9)
**PPA (nM, Median (min-max))**	NA	NA	4450(310.5-8135)

SD: Standard deviation; NA: Samples not available; n: Number of samples; nd: Not detected as concentration below limit of detection.

Table S6. Glyphosate and its metabolites – AMPA and PPA levels in food and water samples in northern and central provinces

Sample type (Single samples unless noted)	Glyphosate	AMPA	PPA
**Northern Areas**			
Chiang Mai Province			
Crab Paste, sample 1 (µg/g)#	127	NA	NA
Crab Paste, sample 2 (µg/g)#	354	NA	NA
Fermented pork (µg/g)#	28.0	NA	NA
Fermented fish sauce (µg/g)#	23.0	NA	NA
Tap water (n=3) (µg/L)#	0.47 (mean)	NA	NA
Bottled water (n=3) (µg/L)#	nd	NA	NA
Water Samples (µg/mL)			
-Bottled water (n=6)	0.020 ± 0.006*	0.001 ± 0.0001	0.449 ± 0.201
-Tap water (n=6)	0.034 ± 0.010*	0.001 ± 0.0002	1.126 ± 0.221
-Well water (n=1)	0.112	0.003	1.455
Fermented fish (µg/g) (n=10)	22.7 ± 15.9	0.30 ± 0.05	14.800 ± 5.090
Crab paste (µg/g) (n=10)	34.4 ± 18.5	0.20 ± 0.07	16.400 ± 2.810
Sukhothai Province			
Fermented fish Sauce (µg/g)#	170	NA	NA
Fermented fish meat (µg/g)#	165	NA	NA
**Central Areas**			
Mahachai, Samutsakorn provinces			
Fermented fish sauce (µg/g)#	74.9	NA	NA
Fermented fish meat (µg/g)#	74.7	NA	NA

*Mean and standard deviation; #Sample collected in 2020; NA: Details not available; n: Number of samples; nd: Not detected as concentration below limit of detection.

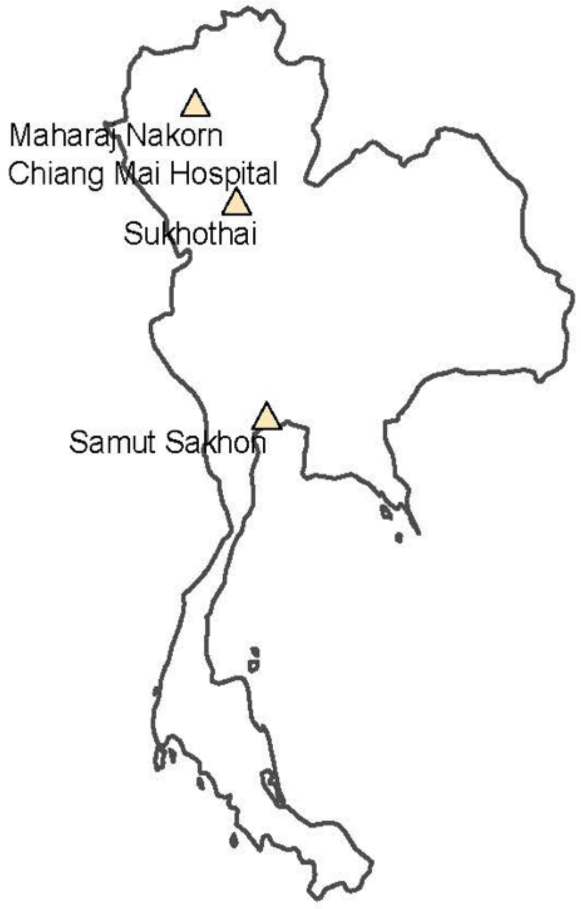

Table S7. Regression model for CLD group compared to Hospital controls

	Estimate	Std.Error	t value	Pr(>|t|)	95% CI
**Glyphosate**	3.48E-04	7.72E-05	4.50E+00	8.79e-06 ***	0.0002-0.0005
**Male vs Female**	4.94E-02	2.35E-02	2.10E+00	0.0365 *	0.0033-0.0955
**AG Vs Non-AG**	6.94E-03	2.75E-02	2.52E-01	8.01E-01	-0.047-0.0609
**Region**	-2.13E-02	1.20E-02	-1.78E+00	7.61E-02 #	-0.0448-0.0022
**Ever Vs Never drinker**	1.27E-02	7.14E-03	1.78E+00	7.60E-02 #	-0.0013-0.0267
**HCV**	6.66E-01	6.21E-02	1.07E+01	< 2e-16 ***	0.5447-0.7882
**HBV**	8.83E-01	2.19E-02	4.03E+01	< 2e-16 ***	0.8402-0.9261
**Raw fish**	-2.57E-02	3.00E-02	-8.58E-01	3.92E-01	-0.0846-0.0331
**Fermented fish**	2.44E-03	2.56E-02	9.50E-02	9.24E-01	-0.0477-0.0526

	Estimate	Std.Error	t value	Pr(>|t|)	95% CI
**AMPA**	1.45E-03	4.52E-04	3.22E+00	0.00217 **	0.0006-0.0024
**Male vs Female**	7.76E-02	6.03E-02	1.29E+00	2.04E-01	-0.0407-0.1959
**AG Vs Non-AG**	-8.93E-02	6.50E-02	-1.37E+00	1.75E-01	-0.2168-0.0382
**Region**	-1.14E-02	3.03E-02	-3.76E-01	7.08E-01	-0.0707-0.048
**Ever Vs Never drinker**	1.06E-02	1.77E-02	5.95E-01	5.54E-01	-0.0243-0.0454
**HCV**	3.76E-01	2.37E-01	1.59E+00	1.18E-01	-0.0887-0.8417
**HBV**	8.86E-01	5.29E-02	1.68E+01	< 2e-16 ***	0.7826-0.9898
**Raw fish**	-1.05E-01	6.47E-02	-1.62E+00	1.10E-01	-0.232-0.0219
**Fermented fish**	4.57E-02	7.00E-02	6.54E-01	5.16E-01	-0.0914-0.1829

	Estimate	Std.Error	t value	Pr(>|t|)	95% CI
**PPA**	1.20E-05	2.88E-06	4.18E+00	3.59e-05 ***	0.0001-0.0001
**Male vs Female**	5.63E-02	2.38E-02	2.37E+00	0.0184 *	0.0097-0.1029
**AG Vs Non-AG**	2.11E-03	2.76E-02	7.60E-02	9.39E-01	-0.052-0.0563
**Region**	-9.57E-03	1.32E-02	-7.23E-01	4.70E-01	-0.0356-0.0164
**Ever Vs Never drinker**	1.20E-02	7.15E-03	1.68E+00	9.42E-02 #	-0.0021-0.0261
**HCV**	7.26E-01	5.89E-02	1.23E+01	< 2e-16 ***	0.611-0.8417
**HBV**	8.68E-01	2.26E-02	3.85E+01	< 2e-16 ***	0.8241-0.9125
**Raw fish**	-3.05E-02	3.02E-02	-1.01E+00	3.12E-01	-0.0897-0.0286
**Fermented fish**	6.28E-03	2.57E-02	2.44E-01	8.07E-01	-0.0442-0.0567

Signif. codes: 0 '*' 0.001 '**' 0.01 '*' 0.05 '#' 0.1 ' ' 1**

Table S8. Regression model for HCC group compared to Hospital controls

	Estimate	Std.Error	t value	Pr(>|t|)	95% CI
**Glyphosate**	-9.98E-04	5.38E-04	-1.85E+00	6.48E-02 #	-0.0021-0.0001
**Male vs Female**	1.33E-02	3.95E-02	3.37E-01	7.36E-01	-0.0642-0.0909
**AG Vs Non-AG**	-2.95E-03	3.93E-02	-7.50E-02	9.40E-01	-0.0801-0.0742
**Region**	-7.21E-02	2.13E-02	-3.38E+00	0.000809 ***	-0.1139--0.0304
**Ever Vs Never drinker**	2.48E-02	1.12E-02	2.23E+00	0.026668 *	0.003-0.0467
**HCV**	6.60E-01	6.98E-02	9.46E+00	< 2e-16 ***	0.5232-0.7969
**HBV**	7.00E-01	4.59E-02	1.53E+01	< 2e-16 ***	0.6105-0.7903
**Raw fish**	2.58E-02	4.10E-02	6.30E-01	5.29E-01	-0.0545-0.1062
**Fermented fish**	5.69E-02	3.83E-02	1.49E+00	1.38E-01	-0.0182-0.132

	Estimate	Std.Error	t value	Pr(>|t|)	95% CI
**AMPA**	1.28E-04	1.52E-04	8.39E-01	4.05E-01	-0.0002-0.0005
**Male vs Female**	1.79E-01	1.13E-01	1.58E+00	1.21E-01	-0.0433-0.4011
**AG Vs Non-AG**	8.91E-02	9.31E-02	9.58E-01	3.43E-01	-0.0933-0.2716
**Region**	-1.41E-01	4.81E-02	-2.94E+00	0.004980 **	-0.2356--0.0471
**Ever Vs Never drinker**	5.18E-02	2.68E-02	1.94E+00	5.87E-02 #	-0.0007-0.1042
**HCV**	6.06E-01	1.64E-01	3.68E+00	0.000564 ***	0.2836-0.9284
**HBV**	5.92E-01	1.01E-01	5.88E+00	3.38e-07 ***	0.3947-0.7895
**Raw fish**	1.11E-01	9.33E-02	1.19E+00	2.41E-01	-0.0721-0.2937
**Fermented fish**	-1.38E-01	1.01E-01	-1.36E+00	1.79E-01	-0.3366-0.0604

	Estimate	Std.Error	t value	Pr(>|t|)	95% CI
**PPA**	5.68E-06	5.25E-06	1.08E+00	2.80E-01	-0.0001-0.0001
**Male vs Female**	1.44E-02	3.97E-02	3.64E-01	7.16E-01	-0.0634-0.0923
**AG Vs Non-AG**	-1.39E-02	3.92E-02	-3.53E-01	7.24E-01	-0.0908-0.063
**Region**	-5.21E-02	2.20E-02	-2.37E+00	0.0185 *	-0.0952--0.009
**Ever Vs Never drinker**	2.42E-02	1.12E-02	2.16E+00	0.0315 *	0.0023-0.0462
**HCV**	6.56E-01	7.04E-02	9.32E+00	<2e-16 ***	0.5184-0.7944
**HBV**	6.92E-01	4.65E-02	1.49E+01	<2e-16 ***	0.6005-0.7829
**Raw fish**	2.78E-02	4.11E-02	6.76E-01	5.00E-01	-0.0528-0.1084
**Fermented fish**	6.05E-02	3.85E-02	1.57E+00	1.17E-01	-0.0151-0.136

Signif. codes: 0 '*' 0.001 '**' 0.01 '*' 0.05 '#' 0.1 ' ' 1**
